# Case Report: Post-Partum SARS-CoV-2 Infection After the First French Uterus Transplantation

**DOI:** 10.3389/fsurg.2022.854225

**Published:** 2022-06-28

**Authors:** Jean Marc Ayoubi, Marie Carbonnel, Niclas Kvarnström, Aurelie Revaux, Marine Poulain, Sarah Vanlieferinghen, Yves Coatantiec, Mathilde Le Marchand, Morgan Tourne, Paul Pirtea, Renaud Snanoudj, Morgan Le Guen, Pernilla Dahm-Kähler, Catherine Racowsky, Mats Brännström

**Affiliations:** ^1^ Department of Obstetrics Gynecology and Reproductive Medicine, Foch Hospital - Paris Ouest Medicine University (UVSQ), Suresnes, France; ^2^ Department of Transplantation, Sahlgrenska Academy, University of Gothenburg, Gothenburg, Sweden; ^3^ Neonatal Care Unit, Foch Hospital, Suresnes, France; ^4^ Department of Clinical Research, Foch Hospital, Suresnes, France; ^5^ Department of Pathology, Hospital - Paris Ouest Medicine University (UVSQ), Suresnes, France; ^6^ Department of Nephrology and Transplantation, Bicêtre Hospital, Le Kremlin-Bicêtre, France; ^7^ Department of Anesthesiology, Foch Hospital - Paris Ouest Medicine University (UVSQ), Suresnes, France; ^8^ Department of Obstetrics and Gynecology, Sahlgrenska Academy, University of Gothenburg, Gothenburg, Sweden; ^9^ Department of Obstetrics, Gynecology and Reproductive Biology, Brigham and Women’s Hospital, Boston, MA, USA; ^10^ Stockholm IVF-EUGIN, Stockholm, Sweden

**Keywords:** uterus transplantation, immunotherapy, COVID-19, SARS-CoV-2, case report

## Abstract

Absolute uterus factor infertility, whether congenital or acquired, renders the woman unable to carry a child. Although uterus transplantation (UTx) is being increasingly performed as a non-vital procedure to address this unfortunate condition, the immunosuppression required presents risks that are further compounded by pregnancy and during the puerperium period. These vulnerabilities require avoidance of SARS-CoV-2 infection in pregnant UTx recipients especially during the third trimester, as accumulating evidence reveals increased risks of morbidity and mortality. Here we describe a successful UTx case with delivery of a healthy child, but in which both mother and neonate developed asymptomatic SARS-CoV-2 infection seven days after RNA vaccination, on day 35 post-partum. Although the patient was successfully treated with a combination therapy comprised of two monoclonal antibodies, this case highlights the challenges associated with performing UTx in the era of Covid-19. More broadly, the risks of performing non-vital organ transplantation during a pandemic should be discussed among team members and prospective patients, weighing the risks against the benefits in improving the quality of life, which were considerable for our patient who achieved motherhood with the birth of a healthy child.

## Introduction

Uterine factor infertility (UFI), a condition estimated to affect 1%–5% of women, is classified either as congenital or acquired. Absolute uterine factor infertility (AUFI), which renders the patient unable to carry a pregnancy successfully, affects approximately 1 in 500 women (1). Among them, Mayer-Rokitansky-Küster-Hauser (MRKH) syndrome with uterine agenesis affects 1 in 4,500 births.

Until recently, the only option for patients with UFI to have biologically related children was through surrogacy, which is banned in many countries and unregulated in others. However, after the first live birth following uterus transplantation (UTx) in 2014 (2), patients suffering from AUFI now have this promising alternative to carry their own child. With 64 published UTx procedures and 24 live births from 15 countries, this transplantation procedure is being increasingly performed, with UTx programs being established by many teams around the world (3).

The SARS-CoV-2 pandemic has significantly impacted solid organ transplantation (SOT) as immunosuppression therapy and associated co-morbidities are responsible for increased risks of severity and mortality (4). Moreover, SARS-CoV-2 infection in the third trimester of pregnancy is associated with an increased risk of maternal and neonatal complications (5). Thus, pregnant UTx recipients who contract SARS-CoV-2 are at potentially high risk of complications. Here we present a patient who underwent successful UTx and delivery of a healthy live born. However, both she and her child contracted the virus in the puerperium period. Despite asymptomatic, our case highlights the risks associated with performing a non-vital organ transplantation procedure in the era of this unprecedented global pandemic.

## Case Report

This study was approved by the Ethics Committee of Paris University in November 2016 (Hôtel Dieu CPP), IRB number IORG0008387.

### The Patient and Her Donor

The patient was a 34-year-old with MRKH syndrome type 1 and no comorbidity. She was identified with MRKH on imaging (the presence of normal ovaries but absent uterus) when she presented with primary amenorrhea at age 17 years of age. She and her 31-year-old husband were first seen in our clinic in 2018 January, expressing the desire to have a biologically related child with UTx. As use of a gestational carrier is not permitted in France, the only option for our couple to achieve this goal was to proceed with UTx after undergoing in vitro fertilization (IVF) and a subsequent thawed embryo transfer.

Our patient’s uterus donor was her 57-year-old mother, who had no severe comorbidity. She was gravida 3, para 3; all three pregnancies were normal and resulted in vaginal deliveries. Both the recipient and donor met the inclusion criteria of our UTx clinical trial (N° NCT03689842) (6).

#### Psychological Counselling and Evaluation

Prior to signing informed consent, the medical team and a multidisciplinary independent committee provided extensive counselling to both patient and mother regarding surgical risks, medical management both prior to and after surgery, and the likelihood of a successful transplantation and birth of a healthy child. Both the patient and her donor had individual interviews with the psychologist to evaluate their ability to participate in the UTx trial and to ensure that there was no undue familial pressure, especially to the mother. After the first and second interview (following a 3-month reflection period), the multidisciplinary uterine transplantation committee decided that the patient, her husband, and mother were eligible to proceed with the treatment.

Psychological support was provided to all three individuals throughout the entire process. Psychological follow up was performed by a dedicated psychologist at each medical appointment using standardized surveys (SF36 for quality of life and HADS for anxiety and depression for the patient, her husband and donor; and Fertiqol for the fertility quality of life for the patient and her husband).

#### Clinical Evaluation

Serology for HHV8 were negative for both donor and patient, and Epstein-Barr virus, cytomegalovirus, HHV6, toxoplasma and VZV were positive for both. Gynecological examination with sampling for sexually transmitted diseases, including high-risk human papilloma virus and herpes simplex virus PCR, were negative. The donor’s cervical smear was normal. The patient had a Polycystic Ovary Syndrome (PCOS) profile. The patient and donor blood groups matched (O+), and they shared eight HLA antigens. Tests for anti-HLA reactive antibodies (with Luminex Single Antigen) and flow cytometry cross-match were negative.

An endometrial biopsy and transvaginal ultrasound revealed a normal uterus in the donor. Conventional angiography successfully confirmed absence of arteriosclerosis and appropriate diameters of the proximal portion of uterine arteries at 1.88 millimeters on the right and 2.25 millimeters on the left. The donor received 3 months of hormone replacement therapy before UTx.

In all, the pre-transplantation process lasted 14 months.

### In Vitro Fertilization

Because preimplantation genetic testing for aneuploidy (PGT-A) is not permitted in France, aggressive ovarian stimulation in two cycles of *in vitro* fertilization (IVF) was performed to ensure enough embryos were frozen for our patient. After the first cycle, 77 oocytes were retrieved from which 34 blastocysts fulfilled our criteria for cryopreservation. However, 29 (85%) showed abnormal developmental kinetics or abnormal developmental phenotypes by time-lapse imaging, despite being suitable for cryopreservation. These abnormalities have been associated with decreased implantation potential (7). We therefore proceeded with a second IVF cycle, from which 44 oocytes were retrieved and 23 blastocysts were frozen; 18 (78%) showed abnormal kinetics and/or abnormalities. In total, 57 blastocysts were frozen, but only 12 revealed no developmental abnormalities. The patient experienced mild ovarian hyperstimulation syndrome in both cycles.

### UTx Surgery

The UTx was performed on March 31, 2019.

#### The Donor

Initial dissection of vessels, achieved using robotic-assisted laparoscopy as used in radical hysterectomy, followed the same steps as described by the Swedish team, including an extensive bilateral ureterolysis (8,9). Final dissection and clamping of vessels was performed after conversion with an infra-umbilical midline incision. The anterior branches of the internal iliac arteries, a patch of the internal iliac vein and uterine branch of the utero-ovarian veins, were clamped bilaterally. On the right side, both uterine and utero-ovarian veins were prepared to allow sufficient venous outflow. On the left, only the uterine vein was prepared. The surgery was completed after 13 h, with blood loss of 150 mL. After two days in the ICU, the donor was discharged on day 11 of post-operative care but required readmission on day 16 for an E. Coli pyelonephritis and sepsis, which was treated with 10 days of iv antibiotics. A left side distal ureteral injury necessitated a ureteral stent on day 17 and six months later, a ureteral reimplantation was necessary because of ureteral stenosis.

The SF36 quality of life survey results for the donor showed a decreased average score 1-month post-hysterectomy compared with that before surgery (60% versus 92%, respectively). However, the score rebounded and had stabilized to 90% by 3 months. Her HADS scores (≤ 7) indicated no sign of anxiety or depression except during the week before the UTx, when her anxiety score was 9.

#### The Patient

Surgical steps for the UTx were as previously described (10). A laparotomy through a sub-umbilical median incision was performed in the patient. The uterine graft was placed in an orthotopic position and bilateral end-to-side vascular anastomosis was performed between segments of the internal iliac vessels of the graft and the external iliac vessels. The surgery proceeded uneventfully (blood loss of 200 mL) and was completed after 6.5 h. The total ischemic time of the uterine graft was 3 h (1 h cold ischemia and 2 h warm ischemia). Following two days in the ICU, the patient was discharged 11 days post-op.

### Immunosuppression Therapy and Patient Follow-Up

**Figure 1:**
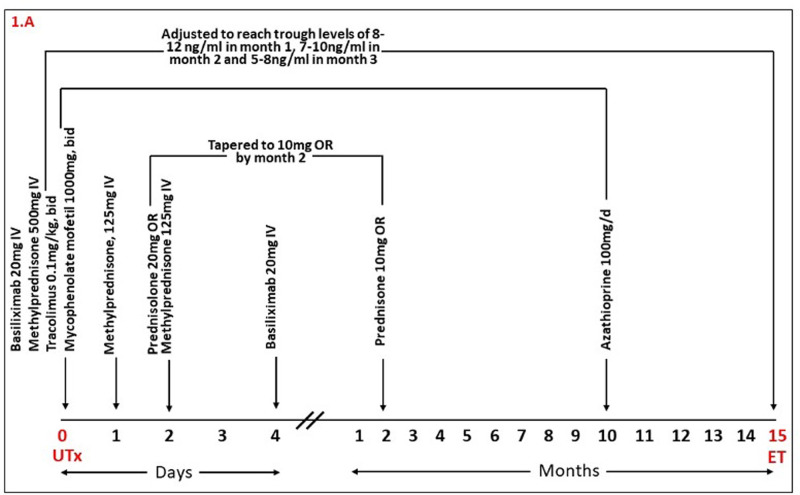
Time-course of immunosuppression therapy during the course of patient treatment from UTx through to embryo transfer. OR, oral; UTx, Uterus Transplantation; ET, Embryo Transfer; d, day.

Administration of immunosuppression therapies are summarized in Figure 1. Immunosuppression was achieved with Basiliximab (Simulect®, Novartis) on days 0 and 4; methylprednisolone (Mylan) on days 0 and 1; and prednisolone thereafter. The patient also received mycophenolate mofetil (MMF; Cellcept®, Roche) and tacrolimus (Adoport®, Sandoz) from day 0, which was adjusted to reach trough levels of 8–12 ng/mL during the first month, 7–10 ng/mL during the second and third month and then 5–8 ng/mL thereafter. MMF was switched to Azathioprine (Imurel®, HAC Pharma) 10 months after UTx and 2 months before the planned embryo transfer, to avoid potentially teratogenic effects of MMF on the fetus.

Acetylsalicylic acid (ASA) and heparin were administered from the day after surgery. Heparin was stopped after 5 weeks, while ASA was stopped 5 days before the C-section, after which it was resumed and will continue until graft removal. As shown in Table 1A, rejection was evaluated with cervical biopsies (according Mölne’s classification) (11) and donor specific antibodies (DSA). Regular clinical evaluation, cervical biopsies, pelvic ultrasonography and blood tests, including assessment of tacrolimus serum levels, were also performed. Frequencies of these evaluations are shown in Table 1B.

**Table 1:** Patient management after UTx and during gestation.

Table 1A Timeline for patient management between UTx and achievement of pregnancy															
	Month Post-UTx Before Pregnancy														
Variable	1	2	3	4	5	6	7	8	9	10	11	12	13	14	15
CBx	4	1	1	1	1	1	1	1	1	1	1	0	0	0	1
U/S	4	1	1	1	1	1	1	1	1	1	1	0	0	0	1
TAC	10	5	4	3	2	2	2	2	2	1	2	0	1	1	1
Visits	3	1	1	1	1	1	1	1	1	1	1	0	1	1	1
DSA	1	0	1	0	0	1	0	0	0	0	0	1	0	0	0
**Table 1B Timeline for patient management during gestation**															
	**Month of Gestation**														
**Variable**	**1**			**2**		**3**		**4**		**5**		**6**		**7**	
CBx	←1→							←0→							
U/S	1			0		1		0		1		1		1	
TAC	2			3		2		2		2		3		6	
Visits	2			1		1		1		2		2		N/A	
DSA	0			0		1		0		0		0		0	

*CBx, Cervical Biopsy; U/S, Ultrasound; TAC, Tacromilus; DSA, Donor Specific Antibodies.*

*Fetal heart monitoring from 29 weeks of gestation.*

The patient’s first menstruation occurred spontaneously 14 days after the UTx with regular menstruation thereafter. The endometrium showed typical changes in thickness, and blood flow velocity waveforms of uterine arteries were within the low to normal range throughout the observation period. No episode of rejection was noticed. Due to the SARS-CoV-2 pandemic and the first French lockdown, embryo transfer was delayed for 3 months, from 12 to 15 months post-UTx.

### Pregnancy

**Figure 2:**
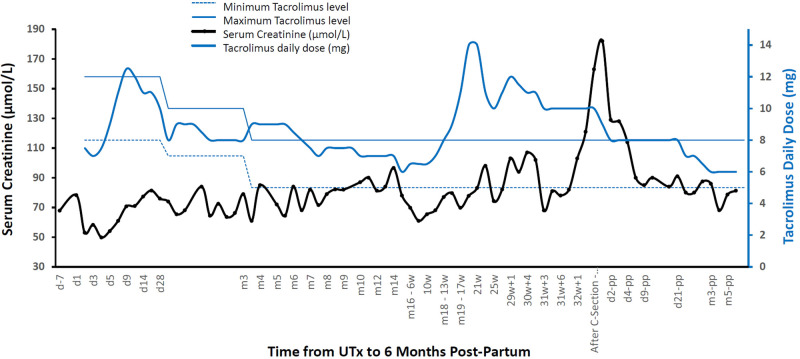
Creatinine and tacrolimus concentrations from the day before UTx until day 6 post-partum. d: day; m: month; w: week of gestation; pp: post-partum. Blue lines represent objective levels of residual tacrolimus level (8–12 ng/mL during the first month, 7–10 ng/mL during the second and third month, and then 5–8 ng/mL).

After transfer of one blastocyst to the uterus with an endometrial thickness of 12 mm, the patient conceived and was treated with oral folic acid, vaginal progesterone (Utrogestan®, Besins international, 400 mg × 2/d, for 12 weeks) and subcutaneous progesterone (Progiron®, Genevier, 25 mg/d for 5 days) (12). During the first trimester, minimal vaginal bleeding occurred (brown discharge, treated with progesterone [25 mg s.c./d] from 8 to 12 weeks of gestation). Fetal growth and blood flow of uterine and umbilical arteries were normal throughout pregnancy. A unilateral adrenal anechoic cyst of diameter 9.7 mm was observed on the 29-week ultrasound and grew to 18 mm by week 32. Creatinine concentrations increased during pregnancy; serum tacrolimus concentrations tended to decrease during the first trimester requiring a doubling in dose thereafter (Figure 2).

**Figure 3:**
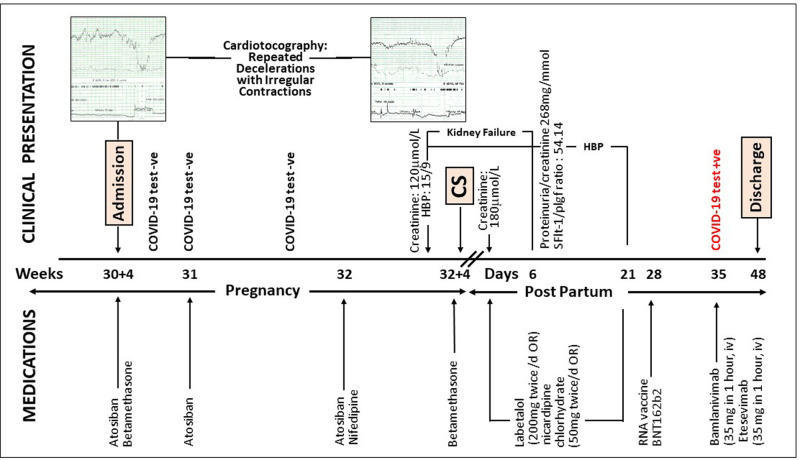
Timeline of clinical presentation and medications during pregnancy and post-partum hospitalization. HBP, High blood pressure; OR, oral; −ve, negative; +ve, positive.

The patient was admitted at 30 weeks and 4 days due to increased uterine contractions, and cardiotocography showed occasional variable decelerations. The cervical length was initially 70 mm and remained stable during the hospitalization. Her medications and clinical presentation during hospitalization are summarized in Figure 3. A subacute Cesarian section was performed at 32 + 4 weeks due to high blood pressure and increased creatine concentrations. Only mild adhesions were observed, and the C-section was uneventful. A healthy girl (1845 g, 42 cm) was delivered with Apgar scores of 7/8/10. The umbilical artery pH was 6.94 with normalization 1 h later. Histological examination showed a normotrophic placenta with disorders of the utero-placental vascularization, but absence of chorioamnionitis. The uterus was retained because the patient expressed her intention to have a second child.

The SF36 quality of life scores for the patient and her husband were good during the entire process (an average of 73% for the patient despite lower scores on physical limitations [60%], and 79% for her husband). Both their HAD scores that revealed no sign of anxiety or depression (scores ≤7). Mean Fertiqol scores were 87.1% for the patient and 79% for her husband.

### Postpartum

Acute renal failure and high blood pressure resolved 1 and 3 weeks, respectively, after delivery (Figure 3). Proteinuria occurred transitorily, and the SFlt-1/plgf ratio (a biomarker of preeclampsia) was positive. The neonate was in good condition and stayed on room air although she had a bilateral inguinal hernia. Both the patient and baby were discharged on day 48 post-delivery. During hospitalization, our patient wore a FFP2/N95 respiratory mask and was only visited by her husband. She and all health care workers regularly used hydro-alcoholic hand rub gel and respected social distancing when feasible.

Per our hospital protocols, three systematic RT-PCR tests were performed on day 0, 2 and 7 of hospitalization for all patients; anyone who tested positive was triaged into a dedicated ward where 70% of healthcare workers had received at least one dose of SARS-CoV-2 vaccination (13). Tests on all three days for our patient were negative and she received one injection of mRNA vaccine (BNT162b2; Comirnati®, Pfizer-BioNTech) on day 28 post-partum. However, both she and her baby tested positive for SARS-CoV-2 infection the day before planned discharge (day 35 post-partum), although both were asymptomatic (her previous test was negative on day 21 post-partum and still positive 45 days post-partum). Azathioprine to the patient was stopped for 4 days and a combination of two monoclonal antibodies, Bamlanivimab and Etesevimab, started. She was finally discharged with her baby 48 days post-partum and tested negative for SARS-CoV-2 1.5 months later. She received her second injection of mRNA vaccine (Comirnati®, Pfizer-BioNTech) 3 months after testing positive; 2.5 months later, her blood SARS Cov-2 antibodies were 8932 Ua/mL. The baby weighed 2976 g at discharge and underwent bilateral inguinal hernia repair at age 3 months without complications; her adrenal cyst disappeared at 4 months.

The patient and donor individually expressed complete satisfaction with their treatment outcomes, culminating in the birth of their healthy daughter/granddaughter. Team members were fully satisfied with the entire process, even though they were stressed by the realization that the first UTx case in France was being performed with the increased risks of complications to the patient if she contracted SARS-CoV-2.

## Discussion

We present a case of post-partum SARS-CoV-2 infection after UTx. Although SOT recipients have been reported to be at higher risk of severe SARS-CoV-2 infection, information is lacking regarding the risk of such infection in UTx patients (14). A recent meta-analysis confirmed that SARS-CoV-2 infection is associated with significantly increased risks of morbidity and mortality in SOT recipients (15), which may partially be explained by the higher incidence of comorbidities in this population. Although our patient had no comorbidities, she was immunosuppressed and thus more susceptible to infection. Taken together, our observations support the need for great caution and extensive counseling to prospective UTx recipients regarding the risks associated with SARS-CoV-2 infection before undertaking this non-vital organ transplantation in the era of the pandemic.

UTx combines two major risk factors: immunosuppressive therapy and pregnancy. The physiological changes that occur during pregnancy increase susceptibility to infection in the puerperium period (16), which may partly explain why our patient tested positive for SARS-CoV-2. The origin of her nosocomial infection remains unidentified, although we assume that incomplete vaccination of our medical health workers was involved. Of note, vaccines in France only became available to the public in January 2021, one month before our patient’s delivery.

Despite some disagreement that immunosuppression is a risk factor of severe COVID-19 disease after SOT (17,18), immunomodulatory therapies have been used to treat severe cases because they reduce the inflammatory response by blunting excessive cytokine release (19). In our case, daily steroids were used prophylactically, yet our patient still developed SARS-CoV-2 infection, albeit asymptomatically. This steroid therapy, combined with treatment of two neutralizing antibodies to SARS-CoV-2 with proven efficacy in reducing viral load in mild to moderate (20,21), as well as asymptomatic (21) SARS-CoV-2 infections, may have collectively reduced the severity of her disease.

Early monoclonal antibody therapy has previously been shown to have favorable outcomes in SOT recipients with minimal adverse effects and no graft rejections (22). Of note, this treatment is recommended by the National Institutes of Health in the United States, for mild and moderate COVID-19 disease in SOT recipients (23). As highlighted in these recommendations, clinicians should pay careful attention to potential drug-drug interactions and overlapping toxicities with immunosuppressants, prophylactic antimicrobials, and other medications when treating SARS-CoV-2 infection in transplantation patients.

The one-week interval between our patient’s first dose of mRNA vaccine and her positive SARS-CoV-2 test was too short to allow an adequate neutralizing antibody response as the IgG response starts after 10 days (24). However, 2.5 months after her second vaccination, her antibody level reached a level sufficient for good coverage, at least for a few months. The antibody response in patients after transplantation is lower than in the general population, which may be associated with their immunosuppressive state (25). A third dose of mRNA vaccine (given at least 4 weeks after the second dose) is currently recommended by the Centers for Disease Control and Prevention for SOT recipients (23).

As with other UTx pregnancies (14), our patient experienced some adverse outcomes, including prematurity and inguinal hernia in the neonate. Obstetricians need to be aware that UTx patients do not feel contractions and so must be especially vigilant regarding the possibility of premature delivery. Our patient also experienced preeclampsia and acute renal failure, which were likely attributable to a renal vasoconstrictive effect of tacrolimus and/or to the uterine graft itself. For all these reasons, intensive follow up of the pregnancy is required by a multidisciplinary team including obstetricians, nephrologists, and specialists in neonate intensive care.

Despite the complications, the extensive surveillance and treatments, the uncertainty of success and the risks associated with a possible SARS-CoV-2 infection, our patient, her husband, and her donor were all satisfied with the entire process, as evidenced by the good quality of life scores and no excessive anxiety. Our observations confirm their determination and highlight the importance of childbearing for those wishing to bear a child who suffer from AUFI.

In conclusion, our patient had a successful UTx which enabled her to carry a child. She suffered premature contractions, high blood pressure and increased creatine levels at 32 + 3 weeks, which together led to premature delivery by C-section at 32 + 4 weeks. She contracted an asymptomatic SARS-CoV-2 infection in the puerperium period, which was successfully treated with a combination of two monoclonal antibodies. The primary take-away points from our case are: (1) UTx is an alternative to childlessness or surrogacy for patients suffering from AUFI; (2) Due to their immunosuppressive state, UTx recipients should be adequately informed of the potential high risk of SARS-CoV-2 complications, which may become more severe with pregnancy; (3) In case of severe cases, the additional risks associated with pregnancy, which is sole goal of UTx, must be extensively covered including the possibility of transplantectomy; (4) UTx recipients who test positive for COVID-19, even when asymptomatic, should be treated with monoclonal antibodies within the first 5 days of infection; (5) Postponement of a non-vital transplantation such as UTx should be discussed with prospective recipients but balanced against their desire to conceive.

## Data Availability

The original contributions presented in the study are included in the article/supplementary material, further inquiries can be directed to the corresponding author/s.
